# Supplementary material for: Personalized neuroimaging reveals the impact of children’s interests on language processing in the brain
Source: Imaging Neurosci (Camb). 2024 Oct 30;2:imag-2-00339. doi: 10.1162/imag_a_00339 (PMC12290543; doi:10.1162/imag_a_00339)
Supplement: Supplementary Material [file imag_a_00339-supp.pdf]

Supplementary Materials

PERSONALIZED LANGUAGE TASK

Supplementary Figure 1: Higher responses to personally interesting narratives in individual participants

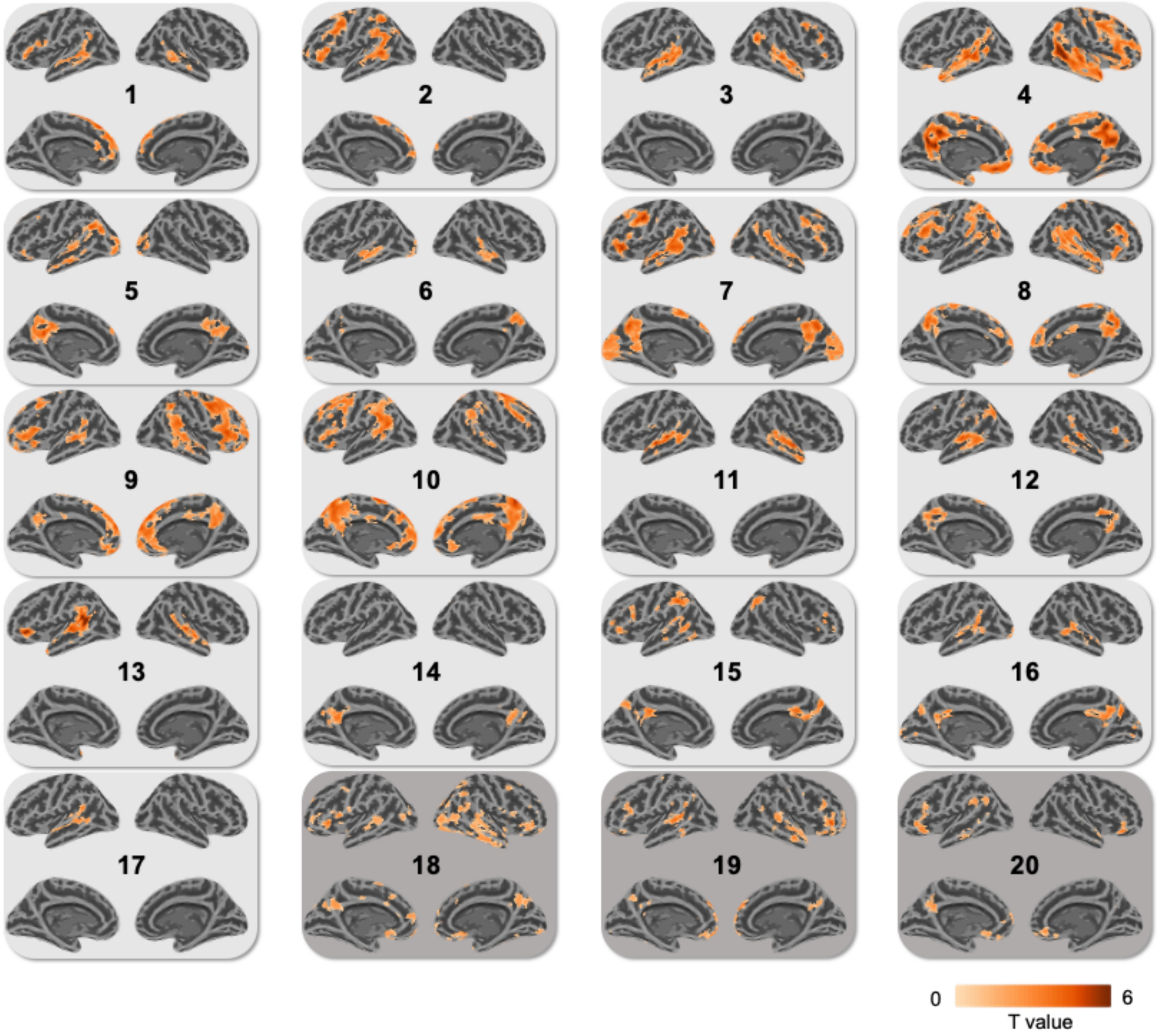

Individual whole-brain responses from 20 participants to INTEREST>NEUTRAL language. N=17 participants visualized at p<0.01, FWE cluster p<0.05. N=3 participants did not show activation at this threshold or had activation that was not visible when projected onto surface space and are therefore visualized at p<0.05 uncorrected (dark grey boxes).

Stimuli characterization.

Non-parametric pairwise comparisons were conducted using Wilcoxon rank-sum tests to identify specific differences between individual subjects' narratives and the neutral narratives. After applying a Bonferroni correction for multiple comparisons (adjusted $p<0.0025$ for 20 comparisons), no significant differences were found for the number of sentences, syllables per sentence, emotional valence, adverb usage, verb usage, and adjective usage across narratives. A few subjects showed significant differences for words per sentence, word count, and word frequency. While noun and proper noun usage were significantly different for several participants when examined separately, these differences were not observed when nouns and proper nouns were combined. Therefore, it is unlikely that the linguistic and paralinguistic features of the narratives were a driving factor in the overall results.

Supplementary Figure 2: Linguistic and paralinguistic features for individual participants' stimuli

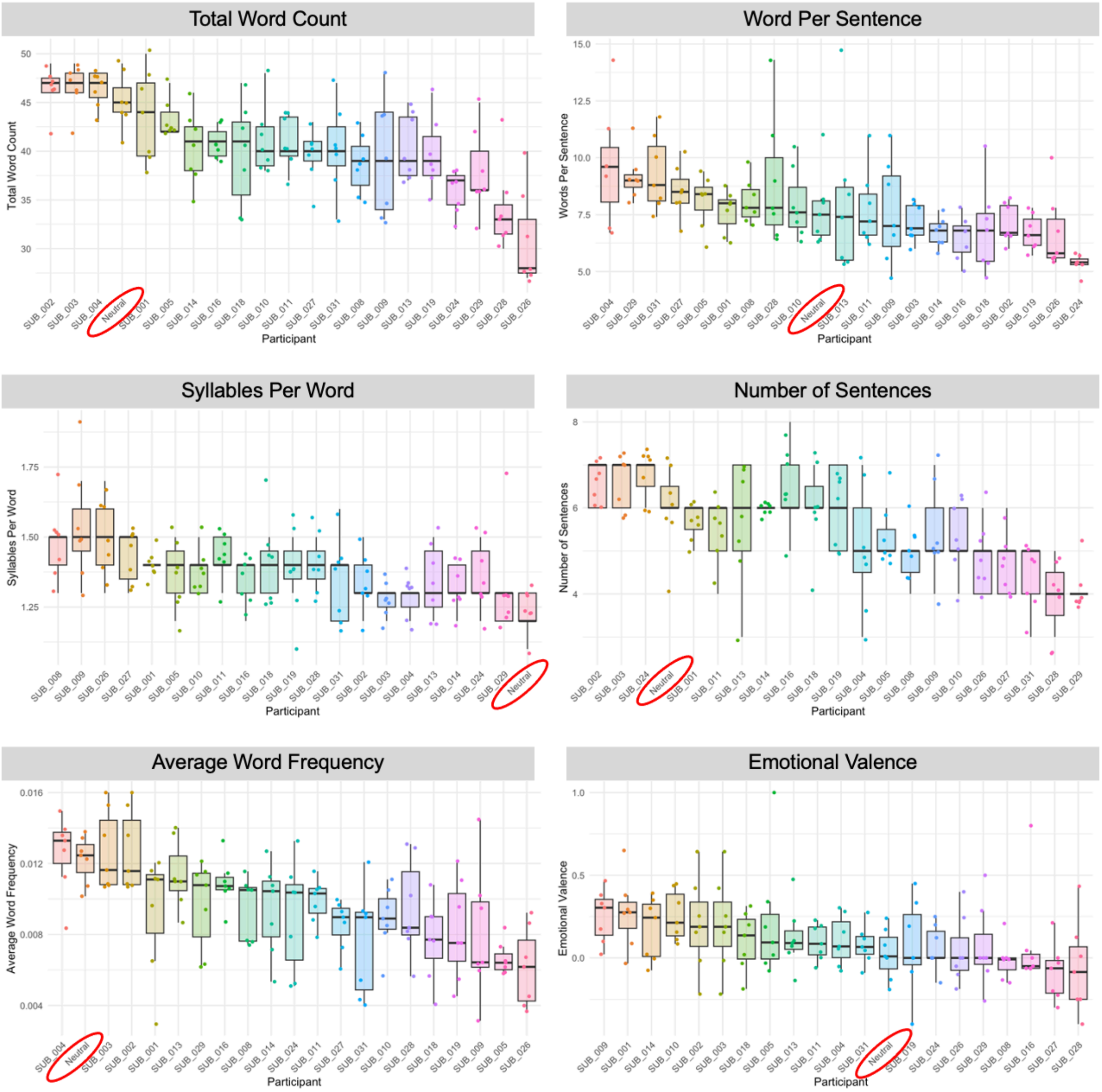

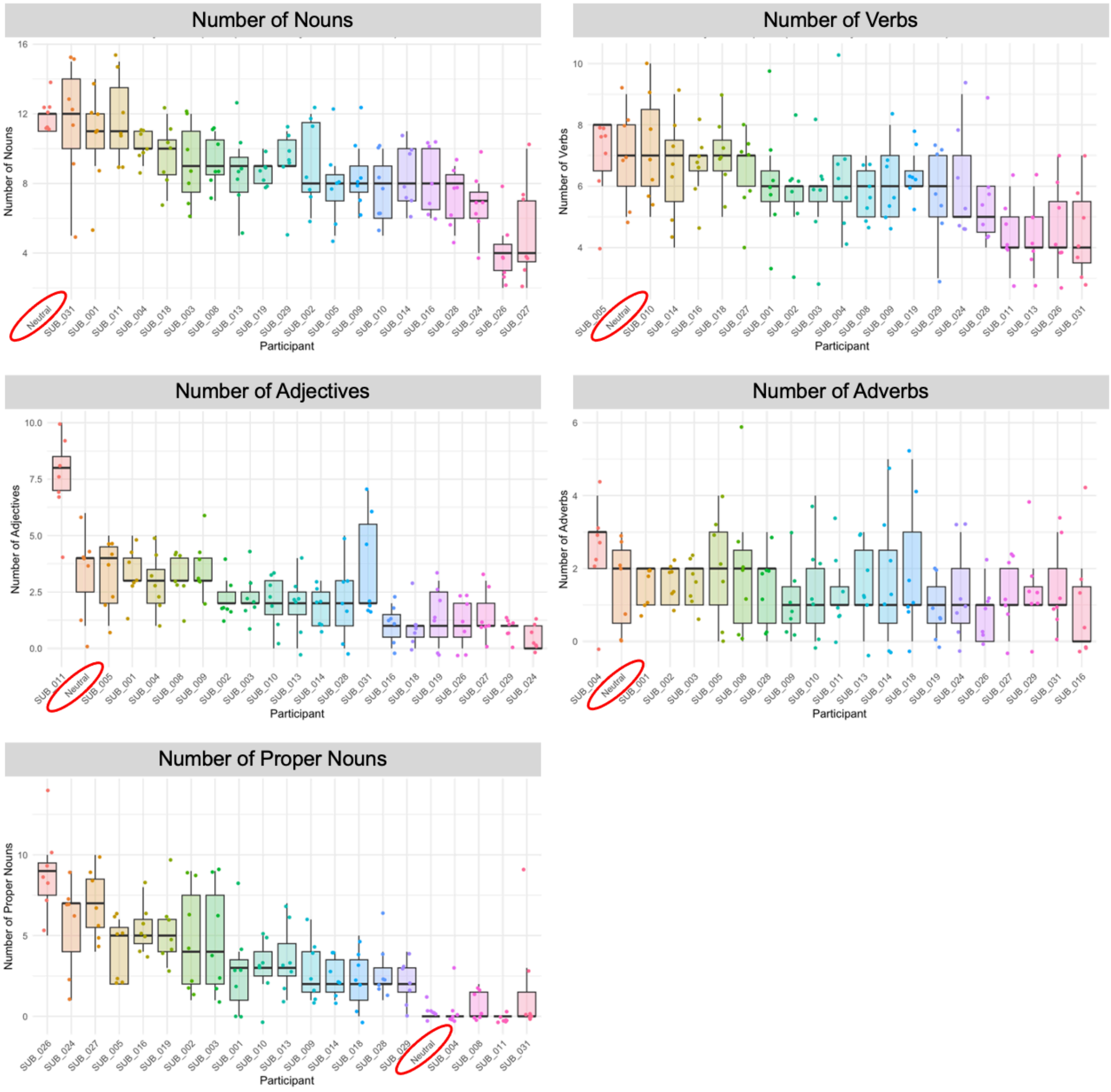

Box plots show linguistic and paralinguistic features by participant, with each narrative represented by a dot. Plots are ordered by median values. The neutral condition is circled in red for reference.

Supplementary Table 1: Linguistic and paralinguistic features for individual participants' stimuli

Subject	Variable	Mean	Standard Deviation	Wilcoxon Statistic	P-value
Neutral	Total Word Count	45.14	2.67	NA	NA
SUB_001	Total Word Count	43.57	4.69	19.5	0.563
SUB_002	Total Word Count	46.43	2.23	33	0.303
SUB_003	Total Word Count	46.57	2.30	33.5	0.273
SUB_004	Total Word Count	46.43	1.90	31.5	0.398
SUB_005	Total Word Count	43.29	1.98	14	0.194
SUB_008	Total Word Count	38.71	3.09	2	0.005
SUB_009	Total Word Count	39.43	6.02	9.5	0.060
SUB_010	Total Word Count	41.14	3.58	7.5	0.034
SUB_011	Total Word Count	41.00	2.71	5	0.014
SUB_013	Total Word Count	40.43	3.46	7	0.028
SUB_014	Total Word Count	40.43	3.69	7.5	0.034
SUB_016	Total Word Count	40.86	1.68	3	0.007
SUB_018	Total Word Count	39.71	5.35	8.5	0.046
SUB_019	Total Word Count	39.71	3.73	6	0.021
SUB_024	Total Word Count	35.86	2.27	0	**0.002**
SUB_026	Total Word Count	30.86	4.95	0	**0.002**
SUB_027	Total Word Count	39.57	2.88	2	0.005
SUB_028	Total Word Count	34.00	4.40	1	0.003
SUB_029	Total Word Count	37.86	4.34	5	0.014
SUB_031	Total Word Count	40.29	4.54	7.5	0.034
Neutral	Words Per Sentence	7.74	1.62	NA	NA
SUB_001	Words Per Sentence	7.66	0.93	27.5	0.748
SUB_002	Words Per Sentence	7.13	0.85	20	0.608
SUB_003	Words Per Sentence	7.16	0.84	21	0.701
SUB_004	Words Per Sentence	9.66	2.61	37	0.125
SUB_005	Words Per Sentence	8.10	1.15	33	0.305
SUB_008	Words Per Sentence	8.09	0.99	32	0.371
SUB_009	Words Per Sentence	7.61	2.26	23.5	0.949
SUB_010	Words Per Sentence	7.96	1.54	25.5	0.949
SUB_011	Words Per Sentence	7.77	1.69	24	1.000
SUB_013	Words Per Sentence	7.97	3.32	22	0.798
SUB_014	Words Per Sentence	6.73	0.64	13.5	0.177
SUB_016	Words Per Sentence	6.47	0.96	12	0.124
SUB_018	Words Per Sentence	6.86	1.96	15.5	0.277
SUB_019	Words Per Sentence	6.71	0.79	13	0.160
SUB_024	Words Per Sentence	5.36	0.39	0	**0.002**
SUB_026	Words Per Sentence	6.71	1.68	12.5	0.141
SUB_027	Words Per Sentence	8.53	1.13	35.5	0.177
SUB_028	Words Per Sentence	8.94	2.84	30.5	0.481
SUB_029	Words Per Sentence	9.17	1.06	41.5	0.034
SUB_031	Words Per Sentence	9.31	1.65	38.5	0.084
Neutral	Syllables Per Word	1.23	0.08	NA	NA
SUB_001	Syllables Per Word	1.40	0.06	47.5	0.003

SUB_002	Syllables Per Word	1.34	0.10	40	0.043
SUB_003	Syllables Per Word	1.29	0.07	34	0.208
SUB_004	Syllables Per Word	1.29	0.07	34	0.208
SUB_005	Syllables Per Word	1.37	0.11	41.5	0.029
SUB_008	Syllables Per Word	1.47	0.13	47.5	0.003
SUB_009	Syllables Per Word	1.54	0.20	47.5	0.003
SUB_010	Syllables Per Word	1.37	0.08	44.5	0.009
SUB_011	Syllables Per Word	1.43	0.08	47.5	0.003
SUB_013	Syllables Per Word	1.34	0.13	37	0.108
SUB_014	Syllables Per Word	1.33	0.08	40	0.042
SUB_016	Syllables Per Word	1.34	0.08	41.5	0.027
SUB_018	Syllables Per Word	1.41	0.15	44.5	0.009
SUB_019	Syllables Per Word	1.39	0.16	41	0.037
SUB_024	Syllables Per Word	1.36	0.11	40	0.043
SUB_026	Syllables Per Word	1.50	0.14	47.5	0.003
SUB_027	Syllables Per Word	1.43	0.10	46	0.005
SUB_028	Syllables Per Word	1.41	0.11	46	0.006
SUB_029	Syllables Per Word	1.31	0.18	31	0.405
SUB_031	Syllables Per Word	1.34	0.15	35.5	0.159
Neutral	Number of Sentences	6.00	1.00	NA	NA
SUB_001	Number of Sentences	5.71	0.49	17	0.296
SUB_002	Number of Sentences	6.57	0.53	33	0.253
SUB_003	Number of Sentences	6.57	0.53	33	0.253
SUB_004	Number of Sentences	5.14	1.46	15.5	0.264
SUB_005	Number of Sentences	5.43	0.79	14	0.179
SUB_008	Number of Sentences	4.86	0.69	8	0.033
SUB_009	Number of Sentences	5.43	1.13	16.5	0.321
SUB_010	Number of Sentences	5.29	0.76	12.5	0.114
SUB_011	Number of Sentences	5.43	0.79	14.5	0.177
SUB_013	Number of Sentences	5.71	1.50	23	0.893
SUB_014	Number of Sentences	6.00	0.00	21	0.593
SUB_016	Number of Sentences	6.43	0.98	29	0.580
SUB_018	Number of Sentences	6.00	1.00	24.5	1.000
SUB_019	Number of Sentences	6.00	1.00	24	1.000
SUB_024	Number of Sentences	6.71	0.49	36	0.116
SUB_026	Number of Sentences	4.71	0.76	7.5	0.028
SUB_027	Number of Sentences	4.71	0.76	7.5	0.028
SUB_028	Number of Sentences	4.00	0.82	3.5	0.007
SUB_029	Number of Sentences	4.14	0.38	4	0.006
SUB_031	Number of Sentences	4.43	0.79	5	0.013
Neutral	Average Word Frequency	0.0122	0.0013	NA	NA
SUB_001	Average Word Frequency	0.0093	0.0034	8	0.041
SUB_002	Average Word Frequency	0.0127	0.0023	27	0.798
SUB_003	Average Word Frequency	0.0127	0.0023	27	0.798
SUB_004	Average Word Frequency	0.0126	0.0022	32	0.371
SUB_005	Average Word Frequency	0.0066	0.0009	0	**0.002**
SUB_008	Average Word Frequency	0.0094	0.0018	6	0.021
SUB_009	Average Word Frequency	0.0080	0.0037	8	0.041
SUB_010	Average Word Frequency	0.0089	0.0018	3	0.007

SUB_011	Average Word Frequency	0.0099	0.0013	5	0.015
SUB_013	Average Word Frequency	0.0114	0.0019	19	0.523
SUB_014	Average Word Frequency	0.0095	0.0026	9	0.055
SUB_016	Average Word Frequency	0.0109	0.0014	12	0.125
SUB_018	Average Word Frequency	0.0077	0.0023	2	0.005
SUB_019	Average Word Frequency	0.0083	0.0028	4	0.011
SUB_024	Average Word Frequency	0.0091	0.0031	9	0.055
SUB_026	Average Word Frequency	0.0061	0.0022	0	**0.002**
SUB_027	Average Word Frequency	0.0086	0.0014	0	**0.002**
SUB_028	Average Word Frequency	0.0095	0.0028	11	0.097
SUB_029	Average Word Frequency	0.0097	0.0025	8	0.041
SUB_031	Average Word Frequency	0.0076	0.0030	2	0.005
Neutral	Emotional Valence	0.0253	0.1543	NA	NA
SUB_001	Emotional Valence	0.2748	0.2168	42	0.030
SUB_002	Emotional Valence	0.2040	0.2804	34	0.250
SUB_003	Emotional Valence	0.2040	0.2804	34	0.250
SUB_004	Emotional Valence	0.1051	0.1499	31	0.443
SUB_005	Emotional Valence	0.2186	0.3725	33	0.307
SUB_008	Emotional Valence	-0.0133	0.1173	17	0.369
SUB_009	Emotional Valence	0.2538	0.1596	43	0.021
SUB_010	Emotional Valence	0.2549	0.1515	42	0.030
SUB_011	Emotional Valence	0.0952	0.1087	32	0.371
SUB_013	Emotional Valence	0.1229	0.1887	32	0.371
SUB_014	Emotional Valence	0.1754	0.1898	36	0.160
SUB_016	Emotional Valence	0.0865	0.3173	21.5	0.749
SUB_018	Emotional Valence	0.1024	0.1788	32	0.371
SUB_019	Emotional Valence	0.0703	0.2837	27	0.797
SUB_024	Emotional Valence	0.0603	0.1381	26.5	0.846
SUB_026	Emotional Valence	0.0438	0.2056	24	1.000
SUB_027	Emotional Valence	-0.0865	0.1724	12.5	0.141
SUB_028	Emotional Valence	-0.0595	0.2802	18	0.443
SUB_029	Emotional Valence	0.0640	0.2505	23.5	0.948
SUB_031	Emotional Valence	0.0795	0.1155	29.5	0.565
Neutral	Number of Nouns	11.86	1.07	NA	NA
SUB_001	Number of Nouns	10.57	2.88	18.5	0.461
SUB_002	Number of Nouns	9.14	2.48	10.5	0.074
SUB_003	Number of Nouns	9.14	2.34	9	0.049
SUB_004	Number of Nouns	10.29	0.76	4.5	0.009
SUB_005	Number of Nouns	8.00	2.24	4.5	0.011
SUB_008	Number of Nouns	9.29	1.50	3	0.006
SUB_009	Number of Nouns	8.43	1.99	4.5	0.011
SUB_010	Number of Nouns	7.57	1.99	0	**0.002**
SUB_011	Number of Nouns	11.71	2.50	21.5	0.740
SUB_013	Number of Nouns	8.71	2.50	6	0.020
SUB_014	Number of Nouns	8.43	1.90	1.5	0.003
SUB_016	Number of Nouns	8.14	1.86	0	**0.002**
SUB_018	Number of Nouns	9.57	1.72	6	0.019
SUB_019	Number of Nouns	8.71	0.76	0	**0.002**
SUB_024	Number of Nouns	6.86	1.86	0	**0.002**

SUB_026	Number of Nouns	4.14	1.95	0	**0.002**
SUB_027	Number of Nouns	5.29	2.81	0	**0.002**
SUB_028	Number of Nouns	7.29	1.60	0	**0.002**
SUB_029	Number of Nouns	9.14	2.04	3	0.006
SUB_031	Number of Nouns	11.43	3.55	26	0.896
Neutral	Number of Verbs	7.00	1.53	NA	NA
SUB_001	Number of Verbs	6.14	2.12	17	0.364
SUB_002	Number of Verbs	5.71	1.50	14	0.192
SUB_003	Number of Verbs	5.71	1.50	14	0.192
SUB_004	Number of Verbs	6.43	1.90	18	0.435
SUB_005	Number of Verbs	7.00	1.53	25	1.000
SUB_008	Number of Verbs	5.86	0.90	13	0.145
SUB_009	Number of Verbs	6.14	1.21	16	0.288
SUB_010	Number of Verbs	7.29	1.80	26.5	0.846
SUB_011	Number of Verbs	4.43	0.98	4	0.009
SUB_013	Number of Verbs	4.43	0.98	4	0.009
SUB_014	Number of Verbs	6.57	1.72	20.5	0.648
SUB_016	Number of Verbs	6.71	0.95	20	0.591
SUB_018	Number of Verbs	7.00	1.29	23.5	0.947
SUB_019	Number of Verbs	6.29	0.95	17	0.359
SUB_024	Number of Verbs	6.14	1.68	17.5	0.384
SUB_026	Number of Verbs	4.71	1.38	6	0.020
SUB_027	Number of Verbs	6.43	1.27	18	0.430
SUB_028	Number of Verbs	5.57	1.72	12.5	0.135
SUB_029	Number of Verbs	5.71	1.50	13	0.145
SUB_031	Number of Verbs	4.57	1.51	6	0.020
Neutral	Number of Adjectives	3.29	2.06	NA	NA
SUB_001	Number of Adjectives	3.29	1.25	21.5	0.738
SUB_002	Number of Adjectives	2.29	0.95	15.5	0.261
SUB_003	Number of Adjectives	2.29	0.95	15.5	0.261
SUB_004	Number of Adjectives	2.86	1.35	19.5	0.555
SUB_005	Number of Adjectives	3.29	1.60	25.5	0.947
SUB_008	Number of Adjectives	3.14	1.07	19.5	0.537
SUB_009	Number of Adjectives	3.57	1.27	22.5	0.841
SUB_010	Number of Adjectives	2.00	1.15	12	0.118
SUB_011	Number of Adjectives	7.57	1.90	46	0.006
SUB_013	Number of Adjectives	2.00	1.29	14	0.188
SUB_014	Number of Adjectives	1.86	0.90	12.5	0.132
SUB_016	Number of Adjectives	1.00	0.82	9.5	0.057
SUB_018	Number of Adjectives	1.00	1.00	9	0.046
SUB_019	Number of Adjectives	1.43	1.27	10	0.068
SUB_024	Number of Adjectives	0.43	0.53	6.5	0.019
SUB_026	Number of Adjectives	1.14	0.90	10	0.067
SUB_027	Number of Adjectives	1.43	1.13	10.5	0.074
SUB_028	Number of Adjectives	2.14	1.77	15	0.242
SUB_029	Number of Adjectives	0.86	0.38	9.5	0.045
SUB_031	Number of Adjectives	3.71	2.21	27.5	0.744
Neutral	Number of Adverbs	1.57	1.27	NA	NA
SUB_001	Number of Adverbs	1.57	0.53	23.5	0.946

SUB_002	Number of Adverbs	1.57	0.53	23.5	0.946
SUB_003	Number of Adverbs	1.57	0.53	23.5	0.946
SUB_004	Number of Adverbs	2.43	1.27	34	0.232
SUB_005	Number of Adverbs	2.00	1.53	29	0.597
SUB_008	Number of Adverbs	2.00	2.08	25.5	0.948
SUB_009	Number of Adverbs	1.14	1.07	19.5	0.553
SUB_010	Number of Adverbs	1.43	1.40	22	0.793
SUB_011	Number of Adverbs	1.29	0.95	21	0.691
SUB_013	Number of Adverbs	1.57	1.13	24.5	1.000
SUB_014	Number of Adverbs	1.71	1.80	24	1.000
SUB_016	Number of Adverbs	1.00	1.53	17.5	0.384
SUB_018	Number of Adverbs	2.00	1.83	26.5	0.845
SUB_019	Number of Adverbs	1.00	0.82	17.5	0.390
SUB_024	Number of Adverbs	1.29	1.25	21.5	0.741
SUB_026	Number of Adverbs	0.71	0.76	14.5	0.206
SUB_027	Number of Adverbs	1.29	0.76	20.5	0.642
SUB_028	Number of Adverbs	1.43	1.13	22.5	0.842
SUB_029	Number of Adverbs	1.43	1.27	22	0.792
SUB_031	Number of Adverbs	1.43	1.13	23	0.894
Neutral	Number of Proper Nouns	0.14	0.38	NA	NA
SUB_001	Number of Proper Nouns	2.86	2.73	41	0.023
SUB_002	Number of Proper Nouns	4.71	3.35	48.5	**0.002**
SUB_003	Number of Proper Nouns	4.71	3.35	48.5	**0.002**
SUB_004	Number of Proper Nouns	0.43	1.13	25	1.000
SUB_005	Number of Proper Nouns	4.00	1.91	49	**0.001**
SUB_008	Number of Proper Nouns	0.71	0.95	32.5	0.228
SUB_009	Number of Proper Nouns	2.86	1.86	48	**0.002**
SUB_010	Number of Proper Nouns	3.00	1.73	45	0.006
SUB_011	Number of Proper Nouns	0.00	0.00	21	0.391
SUB_013	Number of Proper Nouns	3.57	2.15	48.5	**0.002**
SUB_014	Number of Proper Nouns	2.43	1.27	48	**0.002**
SUB_016	Number of Proper Nouns	5.43	1.40	49	**0.001**
SUB_018	Number of Proper Nouns	2.29	1.89	41	0.023
SUB_019	Number of Proper Nouns	5.43	2.30	49	**0.001**
SUB_024	Number of Proper Nouns	5.57	2.94	48.5	**0.002**
SUB_026	Number of Proper Nouns	8.86	2.79	49	**0.001**
SUB_027	Number of Proper Nouns	7.00	2.16	49	**0.001**
SUB_028	Number of Proper Nouns	2.71	1.70	48.5	**0.002**
SUB_029	Number of Proper Nouns	2.14	1.35	44.5	0.008
SUB_031	Number of Proper Nouns	1.71	3.40	29	0.477
Neutral	Number of Nouns (combined)	12.00	1.41	NA	NA
SUB_001	Number of Nouns (combined)	13.43	1.40	40	0.048
SUB_002	Number of Nouns (combined)	13.86	1.35	42	0.027
SUB_003	Number of Nouns (combined)	13.86	1.57	41	0.035
SUB_004	Number of Nouns (combined)	10.71	1.25	10.5	0.071
SUB_005	Number of Nouns (combined)	12.00	2.08	23	0.896
SUB_008	Number of Nouns (combined)	10.00	2.00	9	0.049
SUB_009	Number of Nouns (combined)	11.29	1.38	21	0.686
SUB_010	Number of Nouns (combined)	10.57	2.44	11	0.082

SUB_011	Number of Nouns (combined)	11.71	2.50	20.5	0.642
SUB_013	Number of Nouns (combined)	12.29	1.89	27.5	0.742
SUB_014	Number of Nouns (combined)	10.86	2.54	19.5	0.552
SUB_016	Number of Nouns (combined)	13.57	2.07	36.5	0.134
SUB_018	Number of Nouns (combined)	11.86	2.67	25.5	0.948
SUB_019	Number of Nouns (combined)	14.14	2.12	39	0.068
SUB_024	Number of Nouns (combined)	12.43	1.99	30.5	0.471
SUB_026	Number of Nouns (combined)	13.00	2.58	32	0.360
SUB_027	Number of Nouns (combined)	12.29	2.29	25	1.000
SUB_028	Number of Nouns (combined)	10.00	1.00	3	0.005
SUB_029	Number of Nouns (combined)	11.29	1.38	21	0.686
SUB_031	Number of Nouns (combined)	13.14	1.57	35.5	0.163

Non-parametric pairwise comparisons were conducted using Wilcoxon rank-sum tests for each individual subjects' narratives vs. neutral narratives. Significant differences are bolded, using a Bonferroni correction for multiple comparisons (adjusted p<0.0025 for 20 comparisons).

PERSONALIZED VIDEOS TASK, ANALYSES, AND DISCUSSION

Task design. Participants were asked to passively watch video clips (accompanied by audio) in a block-design paradigm (mirroring the personalized narratives task). The task consisted of two conditions: INTEREST and NEUTRAL. In the INTEREST condition, participants watched the 16-second video clips derived directly from the videos they provided (see **Personalized Stimuli Creation** in Main Text). In the NEUTRAL condition, participants watched 16-second non-personalized videos of nature scenes. Each video was followed by an inter-stimulus rest block of 5 seconds cued by a grey fixation cross (total of 14 videos across two conditions and 15 rest blocks). To confirm that children were attending to the task without imposing significant physical or cognitive demands, we included a low-demand attentional check following each video. An image of a panda appeared on the screen directly after each video for 1.5 seconds, followed by a grey fixation and screen for 0.5 seconds. Children were instructed at the beginning of the study to press a button using their pointer finger via an MRI-compatible button box every time they saw a picture of a panda. Task order was fixed across participants in an [ABAB…] pattern: INTEREST then NEUTRAL.

Analyses. Preprocessing for fMRI data arising from the personalized videos task was identical to that described in methods. Whole-brain analyses were conducted to examine the effect of personal interest videos on the brain. Group-level modeling was performed using SPM12. First-level maps from several contrasts of interest (NEUTRAL, INTEREST, INTEREST > NEUTRAL) were brought up to a second-level analyses wherein one-sample t-tests were used to determine regions for which activation in the condition of interest was greater than baseline. Group maps were thresholded at an uncorrected voxel $p < 0.001$, with a cluster correction for multiple comparisons (FWE < 0.05).

Results. As for the personal interest narrative task, personal interest videos resulted in greater activation in several brain regions when compared to neutral videos. However, unlike the narrative task, many regions showing increased activation were associated with visual rather than language processing including bilateral primary visual cortex, bilateral lateral occipital cortices (LOC), bilateral inferior temporal cortices and fusiform gyrus, bilateral posterior parietal cortex, and right frontal regions.

Discussion. The personalized videos task was designed to be highly naturalistic to reflect children's patterns of brain activation as they authentically engaged with their interest. We solicited timestamps for the videos from families to capture each child's favorite or most salient part of the videos. Unsurprisingly, the INTEREST video clips varied immensely across many dimensions between participants. Some clips had language, some had music; some had humans, some had animals; some had large amounts of movement and action, some had very little movement. The NEUTRAL condition featured nature scenes with no humans, no language, and limited movement (e.g., grass blowing in the wind; snow falling in the forest). Given the variability in the videos, and the many differences between the INTEREST and NEUTRAL conditions on the video task, it is difficult to interpret any specific differences in activation elicited

between the conditions. However, it is notable that brain regions engaged by INTEREST>NEUTRAL on the video task were not the same as those engaged by the personalized language task. While the language task elicited responses in language regions, the videos task elicited responses in primarily visual processing regions (activation in these regions was not observed in for INTEREST>NEUTRAL on the narrative task). Thus, while not the focus of the current paper, the group whole-brain responses on the videos task show that the INTEREST>NEUTRAL contrast in the language task is not identifying a modality-independent set of regions always engaged by any type of interesting materials, otherwise the same network would have been observed for this contrast in the videos task. Similar to what we observed in the language task, an overlapping set of regions are engaged in both the interest and neutral conditions, but interest elicits higher responses in these regions. This further supports the interpretation of a *modulating* effect of interest on modality-specific brain networks.

Supplementary Figure 3: Higher responses to personally interesting videos than generic (nature) videos

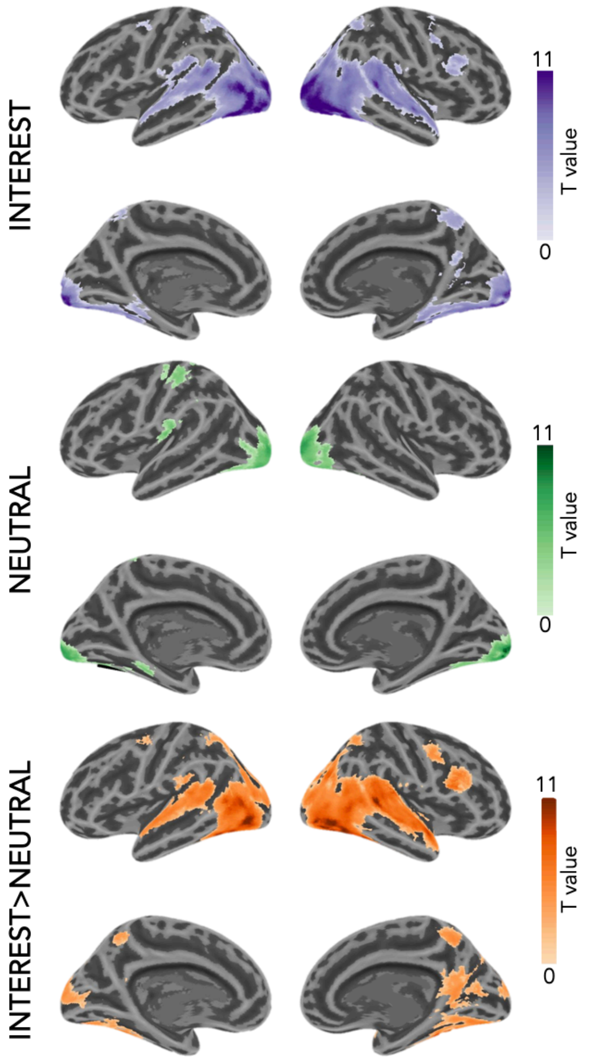

Whole-brain activation maps for the INTEREST, NEUTRAL, and INTEREST>NEUTRAL contrasts from the personalized videos task (threshold: p<0.001, FWE corrected at p<0.05). Color bars reflect t-values.
